# Minimally invasive spleen-preserving surgery to treat primary splenic hydatidosis: short and long-term outcomes: a cohort study

**DOI:** 10.1097/MS9.0000000000002320

**Published:** 2024-07-01

**Authors:** Anas Aljaiuossi, Saleh A. Ba-shammakh, Mohammad Bani Hani, Musab S. Al-A’athal, Yazeed M. Elsobuh, Hashem Abu Sarhan, Raed Mahmoud Ennab, Mohammad Al-Zubi, Mohammad J. Alhwari, Laith G. Al Omari, Feras M. Mohammad, Mohamed S. AL lami, Hammam B. Zeitoon, Saad A. Alomari, Salman M. Ababneh

**Affiliations:** a Faculty of Medicine, Yarmouk University, Irbid; b King Abdullah University Hospital, Faculty of Medicine; c Department of Public Health and Community Medicine, Jordan University of Science and Technology, Irbid; d Department of General Surgery, Ministry of Health, Amman, Jordan; e Department of Ophthalmology, Hamad Medical Corporations, Doha, Qatar

**Keywords:** echinococcus granulosus, hydatid disease, minimally invasive surgery, primary splenic hydatid cyst, recurrence, spleen-preserving surgery

## Abstract

**Background:**

Primary splenic hydatidosis, a rare manifestation of Echinococcus granulosus infection, presents unique diagnostic and therapeutic challenges. This study compares spleen-preserving surgeries with total splenectomy for treating primary splenic hydatid cysts, focusing on short- and long-term outcomes in the Jordanian context, a region particularly affected by this condition.

**Methods:**

This retrospective analysis was conducted on 18 patients diagnosed with primary splenic hydatid cysts at two Jordanian hospitals from January 2015 to June 2021. Selection criteria included confirmed diagnosis and complete medical records. Surgical approaches, including laparoscopic partial splenectomy, cystectomy, and cyst deroofing, supplemented by albendazole therapy, were compared based on patient demographics, symptoms, surgical details, complications, and recurrence rates.

**Results:**

The study group was composed of (*n*=7, 38.9%) male and (*n*=11, 61.1%) female patients, with an average age of 33.7 years. Most presented with left upper quadrant pain. Postoperative complications occurred in 22% of patients, with an 11% recurrence rate during follow-up. No significant statistical difference in recurrence rates was observed between spleen-preserving surgeries and total splenectomy. These findings highlight the efficacy of less invasive, spleen-preserving techniques in managing primary splenic hydatidosis, showing comparable outcomes to total splenectomy with minimal impact on recurrence rates.

**Conclusion:**

Spleen-preserving surgery offers a viable alternative to total splenectomy in treating primary splenic hydatid cysts. This approach maintains immune functionality and reduces septic risks, especially in pediatric patients. The study underscores the importance of individualized treatment approaches and suggests further research with larger cohorts for more comprehensive insights into managing this rare condition. The limitations of this study include its small sample size and retrospective nature.

## Introduction

Highlights

- Spleen-preserving surgery effective for hydatid cysts.
- Outcomes similar to splenectomy with reduced risks.
- Postoperative complications at 22%, 11% recurrence rate.
- Efficient for both adult and pediatric patients.
- Calls for further research, citing small sample size.

Echinococcosis, also known as cystic echinococcosis (CE) or hydatid disease, is caused by the Echinococcus granulosus larva. Hydatid disease primarily affects the liver and lungs, posing a significant health risk^1^. This condition is characterized by the formation of cysts, most notably in these organs. Less frequently, it can be attributed to *E. oligarthrus*, *E. multilocularis*, and *E. vogeli* ^1,2^.

Human infections are mainly due to Echinococcus multilocularis and Echinococcus granulosus, leading to alveolar and cystic echinococcosis, respectively^3^. The global incidence of cystic echinococcosis varies, with rates ranging from 1 to 200 per 100 000 annually^3^. Canids, particularly dogs, serve as primary hosts for the adult form of this tapeworm. In contrast, ungulates, like sheep, often become intermediate hosts after consuming the eggs. Humans are at risk, especially in regions where close contact with these animals is common, potentially becoming accidental intermediate hosts^3,4^.

The disease is a considerable zoonotic concern, especially in regions where sheep farming is prevalent^5,6^. Its high prevalence is notably reported in Middle Eastern countries, such as Jordan, where the close coexistence of sheep, dogs, and humans, particularly among rural and Bedouin communities, increases the risk^7^. The larval form of *E. granulosus*, once hatched, can penetrate intestinal walls, enter the bloodstream, and travel to various organs. The liver is the primary site (75%), with the lungs following (15%), and other organs such as the spleen, kidneys, and others are less commonly affected^8,9^. Primary splenic hydatidosis is rare and often speculated to be due to arterial transmission or transcoelomic spread. It accounts for 0.8–4% of all human hydatid cases and 5.8% of abdominal cases, ranking third in organ involvement after the liver and lungs^10,11^.

The disease’s nature is insidious, with cysts growing slowly over years before becoming detectable^12^. Complications can arise at any stage, including cyst rupture, leading to secondary cyst formation^13^.

The management of primary splenic hydatid cysts remains largely undefined due to its rarity, with no established treatment guidelines. Historically, total splenectomy was the primary approach^14^. However, laparoscopic techniques have recently been favored, as they demonstrate superior outcomes with notably lower complication rates compared to traditional open surgeries^15^. Due to the substantial risks associated with total splenectomy, including the increased risk of severe post-splenectomy infections (OPSI) with a mortality rate as high as 50%, there has been a shift towards spleen-preserving procedures^16^. These less invasive methods, which include techniques like partial cystectomy, cyst unroofing, cystojejunal Roux-en-Y anastomosis and partial splenectomy, aim to retain the spleen’s critical immunological functions while mitigating associated risks^17,18^. Ideal for cases involving superficial, isolated, simple cysts, particularly in younger patients, these techniques have not shown a significant increase in recurrence rates compared to total splenectomy^17,18^. However, they may present challenges such as increased technical complexity and potential for greater bleeding due to the preservation of a larger portion of the spleen^19^.

Historically viewed as non-essential, the spleen is now understood to play key roles in immunity, blood filtration, and iron recycling. The removal of the spleen, particularly in children, can lead to severe complications such as rapid septicemia and increased mortality risk^20–22^. Hence, preventive strategies such as vaccinations and educating patients on infection risks are crucial post-splenectomy^20^.

This study is crucial for understanding splenic hydatid cysts in the Jordanian medical context, underscoring the importance of local data and treatment modalities. It aims to compare the outcomes of spleen-preserving surgeries with total splenectomy, a comparison vital for advancing medical practices in Jordan and other endemic areas.

The study’s main objective was to evaluate the short- and long-term outcomes, with a primary focus on recurrence, of spleen-preserving procedures versus total splenectomy procedures in managing primary splenic hydatid cysts. It aimed to contribute significantly to the field of surgical treatment for this condition, offering insights into the most effective treatment methods that could shape future surgical approaches and patient care strategies in managing hydatid disease.

## Methods

### Participants and settings

This retrospective study involved patients diagnosed with primary splenic hydatid cysts, from January 2015 to June 2021. The study included medical records of 18 patients aged 14 years or older, diagnosed exclusively with primary, non-recurrent splenic hydatid cysts. Exclusion criteria encompassed individuals under 14 years, patients in late pregnancy, those with recurrent cysts, extra-splenic involvement, or less than 2 years of postoperative follow-up.

### Study design and procedure

Diagnostic confirmation of primary hydatid cysts was achieved using computed tomography (CT) scans, abdominal ultrasounds, and KUB CTs. Data collected included demographic information, symptoms, medical history, laboratory findings, diagnostic methods, surgical procedures, postoperative complications, durations of operation and hospitalization, and follow-up data. Cysts were classified according to Gharbi’s and WHO classification systems using CT scans and abdominal US^21,23^ (Tables 1, 2). Surgical options, selected at the time of operation, included laparoscopic partial splenectomy, cystectomy, or cyst deroofing. Patients received three courses of albendazole (15–20 mg/kg, max 800 mg/d): one course before surgery and two after surgery. Follow-up was conducted with an indirect hemagglutination test and ultrasound (US). If the US suggested cyst recurrence, confirmation was obtained through CT scans. Our findings were compared with the existing literature.

**Table 1:** Classification for hydatid cyst type.

Gharbi’s 1981	WHO classification	
Type I	CE1	Active
Type II	CE3a	
Type III	CE2	Transition
	CE3b	
Type IV	CE4	Inactive
Type V	CE5	

**Table 2:** Type of splenic hydatid cyst according to Gharbi’s classification.

Gharbi’s	WHO		Number, *n* (%)
Type I	CE1		2 (11.1)
Type II	CE3a		3 (16.7)
Type III	CE2	3 (16.7)	7 (38.9)
	CE3b	4 (22.2)	
Type IV	CE4		4 (22.2)
Type V	CE5		2 (11.1)

### Surgical technique

Patients eligible for spleen-preserving surgery underwent a thorough preoperative assessment, which included imaging studies to characterize the splenic cysts. Cysts considered for spleen-preserving resection were those located at the periphery of the spleen, had a well-defined wall, and lacked extensive vascular involvement or deep parenchymal connections that might necessitate total splenectomy. All patients received preoperative necessary vaccines 2 weeks prior to surgery, in case total splenectomy was deemed necessary intraoperatively. The position of patients was right decubitus with a 45° tilt, with a sandbag under the lumbar region. A suprarumbilical 11 mm port was inserted using the open Hasson technique; CO_2_ was insufflated to achieve a pneumoperitoneum at 14 mm Hg. Three more working ports were inserted under direct vision: a 10 mm in the left midclavicular line, a 5 mm in the left anterior axillary line at the level of the umbilicus, and a 5 mm in the midline with appropriate triangulation distances. The location of the splenic cyst(s) was determined. Dissection started with Ligasure freeing the splenic attachments, taking down the splenic flexure to varying extents depending on the cyst location. In the case of an upper pole cyst location, the lesser sac was opened and the gastrosplenic ligament was dissected to gain access to the cyst and allow mobilization. After mobilization, gauze soaked with 0.5% cetrimide was packed around the cyst to reduce the chance of dissemination in case of spillage. After that, the cyst was aspirated to collapse and then 0.5% cetrimide was injected inside the cyst—avoiding overdistension of the cyst—and left for 10 min, then aspirated completely. This maneuver was repeated twice. The puncture in the cyst was then widened to allow careful extraction of the germinal membrane and daughter cysts into a preplaced endobag positioned near the cyst to diminish spillage. The cyst wall was then excised and put in the endobag, and the inner epithelial lining was cauterized using monopolar diathermy. The specimen was then sent for histopathology. Suction was further done, and hemostasis was ensured in all cases before a 16 Fr redivac drain was placed near the cyst.

### Outcomes measured

Short-term outcomes included:

- Fluid collection
- Blood transfusion
- Atelectasis
- Surgical site infection
- Hospitalization time

Long-term outcomes focused on recurrence of cysts, assessed through postoperative follow-ups at 3 months, 1 year, and 2 years.

### Statistical analysis

SPSS version 25 (IBM Corp) was utilized for statistical analysis. To explore relationships between variables, descriptive statistics, such as means and standard deviations, were calculated for continuous variables, while frequencies and percentages summarized categorical variables. Inferential statistics, including *t*-tests and correlation tests, were employed to assess the significance of relationships among variables. The analysis was conducted with a significance level set at 5%.

### Ethical considerations

The Institutional Review Board (IRB) of our university approved the study. The study adhered to ethical guidelines, ensuring confidentiality and compliance with healthcare standards. The reporting of this work conforms to the STROCSS criteria^24^.

## Results

Our study involving 18 patients, with a gender distribution of 38.9% male (*n*=7) and 61.1% female (*n*=11), the age range was 15–75 years, with an average age of 33.67 years. The majority (72.22%) of patients presented with left upper quadrant pain (*n*=13), followed by incidental findings (*n*=3), left back pain (*n*=1), and left flank pain (*n*=1). Medical histories indicated one patient with hypertension and another with both hypertension and diabetes mellitus (Table 3).

**Table 3:** Patients demographic and clinical characteristics.

No	Sex	Age	Symptom	Cyst diameter	No. cysts	Operative procedure	Postoperative complications	Hospitalization time (day)
1	F	22	Left upper quadrant pain	9.6	Multiple	LPS	—	5
2	F	22	Left upper quadrant pain	17.1	1	LSC, LSD	—	4
3	F	15	Incidental finding	5.6	1	LPS	—	4
4	F	24	Left upper quadrant pain	13.8	1	LSC, LSD	—	3
5	M	44	Left upper quadrant pain	4.6	1	LSD	—	4
6	F	20	Left upper quadrant pain	13.2	1	LPS, LSC	—	6
7	M	23	Left upper quadrant pain	2.2	1	LSC	—	4
8	M	35	Left upper quadrant pain	4.9	1	LSD	—	4
9	F	26	Left upper quadrant pain	5.9	1	LSD	—	5
10	F	43	Incidental finding	8.2	1	LSD	Fluid collection	9
11	M	38	Left upper quadrant pain	7.7	1	LSD	Blood transfusion	4
12	M	31	Left upper quadrant pain	7.2	1	LSD	—	2
13	F	27	Left upper quadrant pain	7.9	2	LSD	Atelectasis	5
14	F	75	Left back pain	10.2	1	LSD	Surgical site infection and atelectasis	6
15	M	44	Left upper quadrant pain	6.6	1	LSD	—	3
16	F	29	Incidental finding	5.8	2	LSD	—	3
17	M	33	Left flank pain	8.7	1	LSD	—	2
18	F	55	Left upper quadrant pain	9.7	1	LSD	—	4

F, female; LPS, laparoscopic partial splenectomy; LSC, laparoscopic splenic cystectomy; LSD, laparoscopic splenic deroofing; M, male

Preoperative complete blood counts (CBCs) identified eosinophilia in 16.67% of patients (*n*=3). Indirect hemagglutination (IHA) tests were positive in 72.22% of the cohort (*n*=13). Imaging assessments varied, with 10 patients undergoing CT scans, 7 receiving both CT scans and abdominal US, and 1 undergoing CT scans and KUB CT. Gharbi’s Classification revealed the distribution of cyst types as: type I (*n*=2), type II (*n*=3), type III (*n*=7), type IV (*n*=4), and type V (*n*=2) (Table 3).

**Figure 1:**
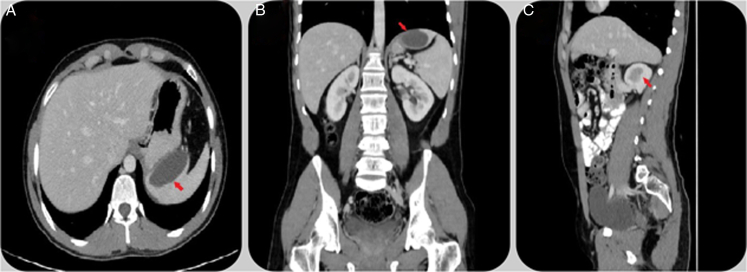
Computed tomography scans of a single splenic hydatid cyst. (A) Axial view showing a unilocular cyst (red arrow). (B) Coronal view with the cyst’s position (red arrow). (C) Sagittal view detailing the cyst’s anteroposterior dimension (red arrow).

**Figure 2:**
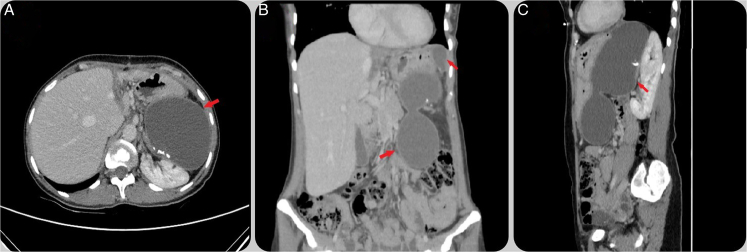
Computed tomography scans of multiple hydatid cysts in the spleen. (A) Axial view of multiple multilocular hydatid cysts (red arrow). (B) Coronal view showing the cysts’ distribution (red arrow). (C): Sagittal view of the cysts’ vertical spread (red arrow).

The average cyst diameter was 8.3 cm, ranging from 2.2 to 17.1 cm. Most patients had a single cyst (*n*=15), while two had two cysts, and one had multiple cysts (Figs. 1, 2). Cyst locations varied across the spleen. Surgical approaches included laparoscopic partial splenectomy (*n*=2), splenic cystectomy (*n*=1), and cyst deroofing (*n*=12), with some undergoing combined procedures. Postoperative complications occurred in four patients, including fluid collection, blood transfusion, atelectasis, and surgical site infections. The average hospital stay was 4.28 days (range: 2–9 days). There were no intraoperative complications or postoperative mortalities (Table 3).

A correlation analysis showed a moderate positive association between operative time and estimated blood loss (EBL) (r=0.498, *P*=0.036). *t*-tests indicated significant gender differences in cyst diameter and hospitalization time, with longer operation times and hospitalization in patients with postoperative complications and those positive for eosinophilia (*P* values: 0.028, 0.036, 0.044, 0.012, respectively).

Postoperative follow-ups at three months, one year, and two years revealed two recurrences (11%), consisting of one intrasplenic cystic lesion and another multiloculated cyst. Both patients presented with left upper quadrant (LUQ) pain at 1 year and 2 years, respectively. Statistical analysis using χ^2^ tests showed no significant association between the types of spleen-preserving surgeries and recurrence, with a *P* value of 0.186 (Table 4). However, the limited number of recurrences observed warrants further investigation in larger cohorts to confirm these findings.

**Table 4:** Spleen-preserving surgery outcomes.

	*n* (%)		
Spleen-preserving surgery	Patient	Short-term complications	Long-term complications
LPS	2 (11.1)	0	1 (50)
LSC	1 (5.6)	0	0
LCD	12 (66.7)	4 (100)	0
LSC, LCD	2 (11.1)	0	1 (50)
LPS, LSC	1 (5.6)	0	0
Total	18 (100)	4 (22.2)	2 (11.1)

LCD, laparoscopic cyst deroofing; LPS, laparoscopic partial splenectomy; LSC, laparoscopic splenic cystectomy.

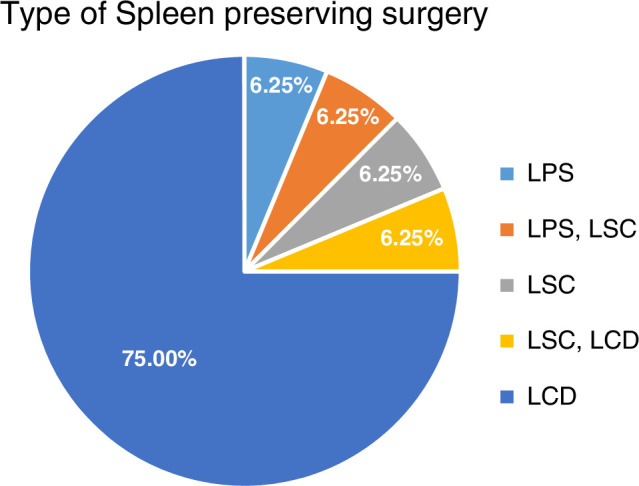

## Discussion

Jordan, located in the Middle East north of the Gulf region, is distinguished for its vast sheep-rearing territories. A prevalent health concern in Jordan is hydatid disease (HD), a condition endemic to this area^25^. The exploration and comprehension of HD in Jordan began to intensify in the late 19th century^26^, with the earliest recorded cases dating to 1966^27^. Further studies have confirmed HD’s endemic status in Jordan, with significant research highlighting an incidence rate ranging from 0.5 to 8.2 cases per 100 000 individuals, averaging 2.9, and identifying the liver and lungs as the most commonly affected organs^28,29^.

Notably, spleen involvement in HD is infrequent, even in endemic regions, accounting for merely 2–3.5% of all cases^30–32^. This rarity is attributable to the distinct dissemination mechanisms of Echinococcus Granulosus eggs, which occasionally bypass hepatic and pulmonary defenses to reach the spleen^33^. Additional, less frequent spread pathways include the portal, lymphatic, and colonic trans-parietal routes^12,34,35^.

Splenic hydatidosis constitutes a mere 0.5–4% of abdominal hydatid disease cases^12,34,35^. Its slow, covert growth—typically 0.3–1 cm per year—often results in prolonged undetected periods, sometimes extending to two decades, thereby contributing to significant clinical latency^36–38^. In our study, 17% of patients were asymptomatic, discovered incidentally during unrelated evaluations. The average age at diagnosis was 33.7 years, with primary symptoms such as left upper quadrant abdominal pain and a palpable mass occurring in 72% of cases.

HD can evolve into complex forms. Complications such as cyst infection, pressure effects, fistula formation, or rupture into adjacent organs can manifest in varied symptoms, including fever, dyspepsia, and anaphylactic shock^33,39–42^.

Diagnostic approaches typically involve routine blood tests, which often yield normal results. Serological tests, such as hydatid immunophoresis and ELISA, demonstrate sensitivity rates between 85 and 90%^33,35,43,44^. However, their efficacy in diagnosing extrahepatic localizations is limited to 65%^19,45^. Our study identified a 28% false-negative rate in indirect hemagglutination tests, suggesting their limitations as sole diagnostic tools.

For imaging, ultrasound and CT scans are pivotal, with ultrasound recommended as the primary examination due to its high sensitivity^19,46^. CT scans, offering even higher sensitivity, are essential for detailed assessment of the cyst’s characteristics^19,46^. Our study indicates that a combination of ultrasonography and CT scans yields a diagnostic accuracy of 100%^47^.

Treatment strategies for splenic hydatid cysts vary between conservative management and surgical intervention. Small, asymptomatic cysts may be managed with anthelmintic drugs, while surgical options, including aspiration and total splenectomy, are primary for more significant cases^39,43,49^. However, there is no consensus on the optimal surgical approach. Surgeons often opt for surgery in symptomatic or larger cysts, mainly due to the risk of complications such as spontaneous rupture^43^. Total splenectomy is typically favored for larger, multiple, or symptomatic cysts, particularly those in central or hilar locations or with concurrent involvement of other organs^33,43^. Despite its efficacy, total splenectomy carries a notable risk of sepsis-related mortality, 4% in children and 1.9% in adults, leading to an increasing preference for spleen-saving surgical procedures^17^.

Spleen-saving techniques, suitable for small, solitary cysts in peripheral locations, include partial splenectomy, enucleation, deroofing with omentoplasty, and internal or external drainage^17,38^. These techniques are complemented by Albendazole-based perioperative therapy, aimed at reducing cyst volume and sterilizing disseminated protoscoleces^17,47^.

Total splenectomy, though technically challenging due to risks like adhesions from chronic pericystic inflammation and potential injury to adjacent organs, stands out as a definitive intervention for preventing recurrences and complications associated with residual cavities in splenic pathologies^33,39,41,48^. Despite its effectiveness, this procedure harbors inherent risks, including overwhelming post-splenectomy infections (OPSI) and thromboembolic events, with mortality rates of 1.9% in adults and 4% in pediatric patients^19,39,49^. Total splenectomy is recommended in specific circumstances such as multiple cysts, spleens with pathological changes, ambiguous protruding domes, centroparenchymal cysts, cysts proximal to the splenic hilum, and cases with significant surrounding organ adhesions^19,33,40,45,50^. It is particularly advocated for managing giant splenic cysts that compromise over 75% of the spleen parenchyma, where substantial parenchymal damage and reduction are observed due to pericystic fibrosis^19,41,45^.

Spleen-preserving surgery emerges as a preferable choice for polar cysts with accessible domes or in scenarios where adhesions elevate the risk of total splenectomy^45^. This approach, while having a lower hemorrhage risk compared to partial splenectomy, is not free from risks, such as postoperative suppuration stemming from residual cavities^51^. Nonetheless, spleen-preserving surgeries, including partial or hemi-splenectomy, are infrequently employed in cases of splenic hydatidosis, with only 24% of 333 surgically managed cases opting for these techniques^42,52–55^. Partial cystectomy remains a preferred method, although laparoscopic partial splenectomy is limited by risks such as intra-abdominal contamination and cyst rupture. Interestingly, laparoscopic partial splenectomy is contemplated for pre-cancerous and cancerous lesions, indicating a potential for broader application in parasitic and oncological contexts^52,55,56^.

Comparing the outcomes of partial splenectomy for hydatidosis with other indications such as hereditary spherocytosis reveals distinct challenges, especially in pediatric surgeries. In children, longer operative times are common, and although morbidity rates are low, significant complications can arise in adults, including hemorrhage and infarction necessitating conversion to total splenectomy^52–54,57,60^. Spleen-preserving surgeries offer the advantage of maintaining immune function and reducing septic postoperative risks. Notably, partial splenectomy, suitable for polar cysts, demands the preservation of at least 25% of splenic tissue but is associated with considerable hemorrhagic risk, particularly in adult patients^37,43,61^. Furthermore, longer hospital stays are often observed when conservative surgical methods encounter complications^37^.

The laparoscopic approach is increasingly favored due to its minimally invasive nature and effective outcomes. However, it bears risks such as accidental cyst rupture and anaphylactic shock, potentially leading to spillage of cystic fluid and secondary peritoneal echinococcosis. This technique is particularly suitable for small splenic hydatid cysts but is generally not recommended for multiple or infected cysts^19,45,51^. Its limitations also encompass the high costs of training and equipment, as well as the necessity for advanced surgical skills.

Puncture Aspiration Injection and Reaspiration (PAIR) has emerged as a promising therapeutic strategy, increasingly favored for its minimally invasive approach. Esteemed for its safety profile, PAIR facilitates reduced hospitalization and mitigates morbidity, markedly outperforming spleen-preserving surgeries and total splenectomy in these aspects^50^. It serves as a formidable option for patients contraindicated for general anesthesia^19,45^. Nevertheless, its applicability remains confined to uncomplicated type I or II cysts, preferably smaller than 5 cm in diameter^31,41,45^. A significant gap in the literature persists, as no comprehensive studies have yet juxtaposed its long-term complications, notably recurrence rates, against other surgical interventions.

In the realm of short-term complications, our study found no significant differences in postoperative hospitalization duration or the emergence of other complications when comparing spleen-preserving procedures with total splenectomies^17,36,43^. It is noteworthy that 22% of the patients (*n*=4) encountered postoperative complications, which varied from fluid collection and atelectasis to surgical site infections. Furthermore, there was a case where a patient necessitated a postoperative blood transfusion owing to intraoperative bleeding. These complications were effectively managed and resolved. In contrast, several studies highlight the advantages of spleen-preserving surgeries, especially in laparoscopic techniques. These studies underline the less invasive nature of spleen-preserving surgeries, faster recovery periods, and lower rates of postoperative complications compared to total splenectomy^62–64^.

In terms of long-term outcomes, research suggests no significant disparity in recurrence rates between spleen-preserving surgeries and total splenectomy^17,36,43^. Our study noted a recurrence in a mere 2 cases (11%, *n*=2), a statistically insignificant figure. However, other studies indicate lower rates of complications and recurrence^62–64^, a finding particularly crucial in pediatric cases due to the spleen’s vital immunological functions.

To conclude, spleen-preserving surgery emerges as a beneficial treatment alternative for splenic hydatid cysts. It upholds immune functionality and minimizes septic risks, especially in pediatric patients. Customizing the surgery based on individual patient needs and cyst characteristics is imperative for optimal outcomes.

Our study, albeit enlightening, is not without inherent limitations that merit acknowledgement. Firstly, the limited sample size, consisting of only 18 patients, cautions against the broad extrapolation of our findings to a wider population. Secondly, being conducted exclusively at two centers, the generalizability of our findings to diverse geographical or clinical settings may be constrained. Furthermore, the absence of a randomized control group within our study framework restricts our ability to definitively attribute outcomes solely to the treatment methods, as other variables could potentially influence the results. Given the rare occurrence of primary splenic hydatid cysts, our study, though offering valuable insights, underscores the pressing need for larger cohorts to corroborate our findings. Lastly, the limited number of recurrences observed underscores the necessity for further exploration through more extensive studies. To enhance the transparency and integrity of our research, we emphasize the importance of presenting our findings honestly and advocating for more comprehensive future studies to fortify these initial observations.

In conclusion, this study comprehensively compares spleen-preserving surgeries with total splenectomy for treating primary splenic hydatid cysts. We demonstrate that techniques like laparoscopic partial splenectomy, cystectomy, and cyst deroofing are effective, with similar recurrence rates to total splenectomy.

These findings are vital for guiding clinical decisions in managing primary splenic hydatidosis. However, given the study’s limited sample size, further research is essential, especially to understand the long-term impacts of these surgical options. Future studies should focus on larger cohorts to validate our results and explore broader implications for patient health.

## Ethical approval

Ethical approval for this retrospective study was duly obtained in accordance with standard research ethics guidelines. The study was reviewed and approved by the Institutional Review Board (IRB) of Al-Yarmouk University. The ethical approval encompasses the review and analysis of patient records under the specified conditions of confidentiality and data protection.

## Consent

Written informed consent was obtained from the patient for publication and any accompanying images. A copy of the written consent is available for review by the Editor-in-Chief of this journal on request.

## Source of funding

This research did not receive any specific grant from funding agencies in the public, commercial, or not-for-profit sectors.

## Author contribution

A.A. and S.A.B.: both authors were significantly involved in the study design, equally contributing to the conceptualization, planning of the research, and manuscript drafting. M.B.H. and M.S.A.-A.: participated in data analysis and interpretation, providing critical insights for the study's findings and manuscript drafting. Y.M.E., H.A.S., R.M.E., M.A.-Z., M.J.A., L.G.A.O., F.M.M., M.S.A., H.B.Z., S.A.A., S.M.A.: engaged in data collection and contributed to the manuscript drafting process. All authors have reviewed and approved the final draft of the manuscript, affirming their agreement with its content and conclusions.

## Conflicts of interest disclosure

The authors have no conflicts of interest to disclose relevant to this article.

## Research registration unique identifying number (UIN)

Our research was rigorously registered, aligning with the ethical guidelines outlined in the World Medical Association's Declaration of Helsinki (2013), which governs the conduct of research involving human subjects. The registration details are as follows:

- Registry: ClinicalTrials.gov
- Registration ID: NCT06289816
- Registration URL: https://clinicaltrials.gov/study/NCT06289816

## Guarantor

Saleh A. Ba-shammakh.

## Data availability statement

Data are available from the corresponding author upon reasonable request and with permission of the respective hospitals.

## Provenance and peer review

Not commissioned, externally peer-reviewed.
